# Combined high rates of alternative breeding strategies unexpectedly found among populations of a solitary nesting raptor

**DOI:** 10.1002/ece3.70190

**Published:** 2024-08-19

**Authors:** Robert N. Rosenfield, Sarah A. Sonsthagen, William E. Stout, Timothy G. Driscoll, Andrew C. Stewart, Paul N. Frater, Sandra L. Talbot

**Affiliations:** ^1^ Department of Biology University of Wisconsin‐Stevens Point Stevens Point Wisconsin USA; ^2^ U.S. Geological Survey, Nebraska Cooperative Fish and Wildlife Research Unit, School of Natural Resources University of Nebraska Lincoln Nebraska USA; ^3^ Oconomowoc Wisconsin USA; ^4^ Urban Raptor Research Project Grand Forks North Dakota USA; ^5^ Cobble Hill British Columbia Canada; ^6^ Center for Limnology University of Wisconsin‐Madison Madison Wisconsin USA; ^7^ Northwestern Institute of art and Science Anchorage Alaska USA

**Keywords:** *Accipiter cooperii*, alternative mating strategies, conspecific brood parasitism, extra‐pair paternity, floaters

## Abstract

Social monogamy is the prevalent mating system in birds, but alternative strategies of extra‐pair paternity (EPP) and conspecific brood parasitism (CBP) occur in many species. Raptors are virtually absent in discussions of broad taxonomic reviews regarding EPP and CBP likely because these strategies are mostly absent or at low frequency; CBP is unreported in solitary nesting raptors. In contrast, we found high frequencies of EPP (16%–31%) and CBP (15%–26%) nests among three populations of Cooper's Hawks (*Accipiter cooperii*) across the northern breeding range of this solitary nesting, socially monogamous species. EPP and CBP combined occurred in 42%–46% of all nests among populations and hence unexpectedly were nearly equivalent to proportions of genetically monogamous nests. Select covariates failed to predict presence of EPP and CBP in part because virtually all extra‐pair adults were uncaught and likely were floaters. We found no support for the hypothesis that territorial females traded copulations for food to maximize energy intake for increased production. Our unique discoveries enhance knowledge of the extent and diversity of alternative breeding strategies among groups of avian and other animal species.

## Graphical abstract

Raptors are virtually absent in discussions of broad taxonomic reviews regarding extra‐pair paternity (EPP) and conspecific brood parasitism (CBP) likely because these strategies are mostly absent or at low frequency. In contrast, we found high frequencies of EPP (16%–31%) and CBP (15–26%) per nest among three populations of Cooper's Hawks (*Accipiter cooperii*) across the northern breeding range of this solitary nesting, socially monogamous species. Our unique discoveries enhance knowledge of the extent and diversity of alternative breeding strategies among groups of avian and other animal species.

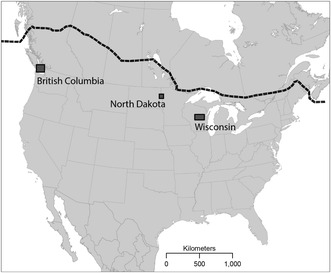

## 1 INTRODUCTION

Mating systems are fundamental to our ecological and evolutionary understanding of animal populations because they influence levels of genetic diversity, gene flow, and population structure (Gunn et al., 2022). Although social monogamy with biparental care is the most prevalent mating system among birds, inferred in 9456 species worldwide (Cockburn, 2006), genetic promiscuity via extra‐pair paternity (EPP, or genetic polyandry) is a common alternative reproductive strategy in socially monogamous birds. In fact, extra‐pair copulations (EPCs) occur in ca. 90% of all bird species (Griffith et al., 2002), particularly in altricial passerines (Brouwer & Griffith, 2019).

Compounding our ability to understand the role of alternative reproductive strategies in birds is the occurrence of conspecific brood parasitism (CBP), whereby females lay eggs in the nests of other conspecifics; CBP occurs in about 2% of all bird species, especially in precocial, colonial, or cavity‐nesting birds (Lyon & Eadie, 2008; Yom‐Tov & Geffen, 2017). Unfortunately, there is an absence of general ecological drivers that explain variation in the presence of extra‐pair young (EP) from both EPP and CBP across and among populations of species (Brouwer & Griffith, 2019; Lyon & Eadie, 2008). Notably, few studies have investigated the occurrence of EPP and CBP among intra‐specific populations across a large geographic scale where varying local conditions may produce disparate ecological drivers (Brouwer & Griffith, 2019).

Raptors are virtually absent in discussions involving broad taxonomic reviews of the occurrence of EPP and CBP in birds (Brouwer & Griffith, 2019; Lyon & Eadie, 2008) perhaps because most raptor studies reveal no or low rates of a maximum of about 7% of nests with CBP and 13% with EPP (Costanzo et al., 2020; Villarroel et al., 1998). CBP has been documented in only two raptors, the Lesser Kestrel (*Falco naumanni*; Costanzo et al., 2020) and the Red‐footed Falcon (*F. vespertinus*; Magonyi et al., 2021); these species are colonial breeders. The non‐existent or low rates of EPP in raptors are intriguing given that males are at high risk of being cuckolded because mate guarding, the common avian strategy to assure paternity, is compromised when territorial male raptors leave their non‐hunting female mates unattended before egg‐laying to capture food for them (Mougeot, 2004; Newton, 1979). Further, paired females may engage in EPCs during extra‐territorial visits (e.g., Poole, 1985; Rosenfield, 2017).

By contrast, in a colonizing population of Cooper's Hawks (*Accipiter cooperii*; a biparental, solitary breeder), it was reported that 34% of all broods had at least one EPP nestling (Rosenfield et al., 2015). This finding is discordant with the hypothesis that in species with high parental investment like Cooper's Hawk, females should avoid EPCs (Ledwoń & Szczy, 2021). It was hypothesized that the high rate of EPP was due to the strong association between courtship feeding and copulations (not found in other raptors), and a high nesting density of breeders (Rosenfield et al., 2015).

The socially monogamous Cooper's Hawk breeds across the contiguous United States into southern Canada and central Mexico (Rosenfield, 2018). High nesting densities and productivity indices for Cooper's Hawks co‐occur in urban and rural populations of this avivorous, territorial species (Rosenfield et al., 2020). Breeding males almost exclusively provide food for themselves, their mates, and offspring from pre‐incubation through most of the nestling and post‐fledging periods. Intra‐sexual aggression occurs in territorial adults via physical attacks and killing of territory intruders (Rosenfield et al., 2020).

**FIGURE 1:**
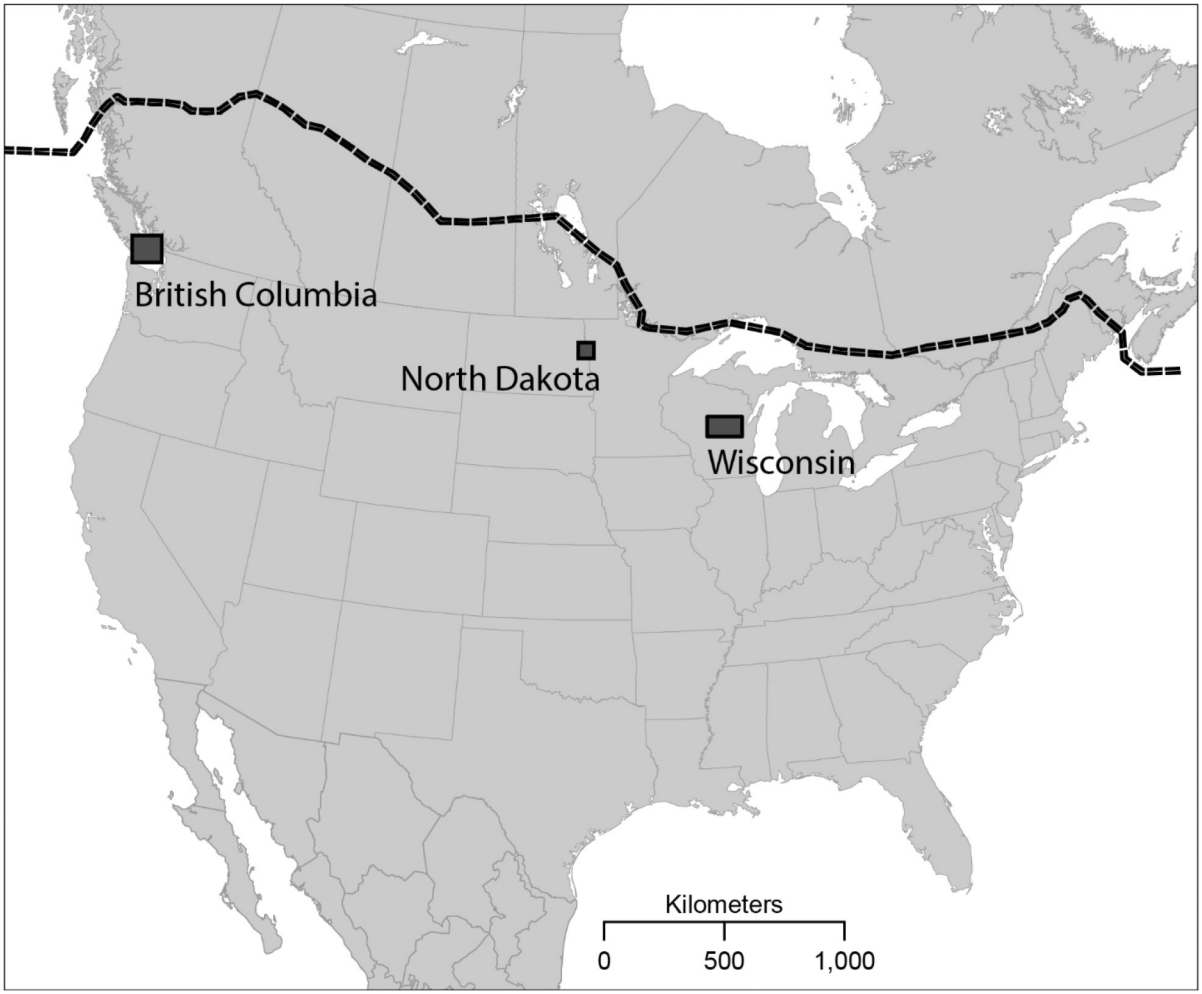
The northern extent of Cooper's Hawk (*Accipiter cooperii*) distribution (dashed line) and location of study sites.

Given the afore‐stated paucity of intra‐specific, large spatial scale studies of extra‐pair biology in birds, herein we examine EPP and CBP rates, the latter surprisingly discovered, among three Cooper's Hawk populations across their northern breeding range (Figure 1; Stout & Rosenfield, 2010, Rosenfield, 2018). Nesting densities at sampled populations are similar to those estimated in the colonizing population located in Milwaukee (southeast Wisconsin, United States of America). Following the results from the Milwaukee population, we made a series of specific predictions. (1) EPP rates among study sites will be similar to those reported in the Milwaukee population. (2) Variations in EPP rates within study sites will not vary by male mass, or female mass or age. (3) EPP rates will vary positively with brood size. (4) Extra‐pair occurrences will predominately be the result of unsampled, apparently unpaired floaters. We were particularly interested in testing the hypothesis that territorial females traded copulations for food to maximize energy intake for increased production as suggested by Rosenfield et al. (2015). We had no predictions regarding CBP because it is unknown to occur in solitary, territorial raptors, and no evidence of CBP was found in the Milwaukee population or (knowingly) elsewhere.

## 2 METHODS

### 2.1 Study areas and populations

Our three study sites spanned ~2700 km across the northern part of Cooper's Hawk's breeding range (43°–48° N, Figure 1). The 89‐km^2^ British Columbia (BC) study site in southern Vancouver Island, British Columbia, Canada (48° N; 123° W) included 19 nests in 19 territories studied in 2011 throughout the city of Victoria with a human population of ~240,000 people (see Rosenfield et al., 2010).

The 37 km^2^ study site in eastern North Dakota (ND; 47° N; 97° W), ~1870 km east of the BC site, included 53 nests in 30 territories in the city of Grand Forks, and the abutting border city of East Grand Forks, Minnesota, 2012–2015. This site has a human population of ~60,000 people (Rosenfield et al., 2010).

The Wisconsin (WI) study site, ~825 km east‐southeast of ND, in central Wisconsin (43° N; 88° W), included 48 nests in 40 territories: 19 nests in 19 rural territories, and 29 nests in 21 urban territories among the cities of Stevens Point (~35,000 people) and Oshkosh (~60,000 citizens) (Rosenfield et al., 2010, 2020). This study site included 35 km^2^ in Stevens Point and 31 km^2^ in Oshkosh. Urban nests were those occurring within the municipal limits of cities; rural nests were those in rural forested tracts, typically ≥16 ha, usually with ≤3 houses within 0.4 km of a nest. A territory was defined as an area centered at nest sites of 800 m in diameter occupied by a breeding adult in one or more years; a territory was reoccupied when a new nest occurred within 400 m of the original nest (Rosenfield & Bielefeldt, 1999). This study site does not include the southeast Wisconsin (Milwaukee) study site of Rosenfield et al. (2015).

### 2.2 Field techniques

Capturing and handling of Cooper's Hawks follow Rosenfield et al. (2015). Briefly, we trapped and individually marked adults with U.S. Geological Survey aluminum and colored leg bands, 2011–2015. Adults were aged by plumage as either one‐year‐old, or birds ≥2 years of age. Body mass of breeding adults (±1 g) was determined at the nestling stage with a balance beam or digital scale; mass of adults at this time is a reliable index for size (Sonsthagen et al., 2012). At least one adult was captured in each territory (Table 1). Nestlings were counted and then marked with U.S. Geological Survey aluminum leg bands.

**TABLE 1:** Number of adults, nests, and nestlings sampled; percentage and number of extra‐pair paternity (EPP) and conspecific brood parasitism (CBP) young and total extra‐pair (EP) young and nests; the percentage and number for the total extra pair (EP) nests; and number of unsampled adults involved in extra pair occurrences in populations of Cooper's Hawks breeding in British Columbia, North Dakota, and Wisconsin within and across years, and all sites combined.

Year	Adults	Nests	Nestlings	EPP	EPP	CBP	CBP	EP young	EP young	EPP nests	EPP nests	CBP nests	CBP nests	EP nests	EP nests	EP adults
	M:F:P^a^	*N*	*N*	%	*N*	%	*N*	%	*N*	%	*N*	%	*N*	%	*N*	M:F
British Columbia																
2011	1:00:18	19	67	11.9^d^	8^b^	14.9^d^	10	26.9	18	15.8^d^	3	26.3^d^	5	42.1^d^	8	2:6
North Dakota																
2012	3:04:09	16	55	14.5	8	5.5	3	20.0	11	31.3	5	6.3	1	37.5	6	7:4
2013	1:03:14	18	63	12.7	8	11.1	7	23.8	15	22.2	4	27.8	5	50.0	9^c^	6:6
2014	0:00:11	11	42	19.0	8	2.4	1	21.4	9	45.5	5	9.1	1	45.5	6	3:2
2015	0:02:06	8	32	9.4	3	12.5	4	21.9	7	25.0	2	12.5	1	37.5	3	4:2
Total	4:09:40	53	192	14.1^d^	27	7.8^d^	15	21.9	42	30.2^d^	16	15.1^d^	8	45.3^d^	24	20:14
Wisconsin																
2012	3:02:04	9	31	19.4	6	6.5	2	25.8	8	44.4	4	22.2	2	66.7	6^c^	5:3
2013	2:00:10	12	47	10.6	5	14.9	7	25.5	12	33.3	4	25.0	3	58.3	7	4:5
2014	2:01:10	13	48	10.4	5	4.2	2	14.6	7	23.1	3	7.7	1	30.8	4	2:3
2015	0:01:13	14	59	13.6	8	1.7	1	15.3	9	28.6	4	7.1	1	35.7	5	4:2
Total	7:04:37	48	185	13.0^d^	24	6.5^d^	12	19.5	36	31.3^d^	15	14.6^d^	7	45.8^d^	22	15:13
All Sites																
	12:13:95	120	444	13.3	59	8.3	37	21.6	96	28.3	34	16.7	20	45.0	54	37:33

*Note*: There were no significant differences among all three study sites in total proportion of: EPP young /nest (*X* ^2^_2_ = 0.17, *p* = .92), CBP young/nest (*X* ^2^_2_ = 3.81, *p* = .15), EPP nests (*X* ^2^_2_ = 1.07, *p* = .59), CPB nests (*X* ^2^_2_ = 1.08, *p* = .60), and EP nests (*X* ^2^_2_ = 0.31, *p* = .98).

^a^ Number of adults sampled in territories is reported as males and females captured without mates and paired adults. Adults without associated young were not included.

^b^ Only one male was identified as an EPP sire. A territorial male in British Columbia 2011 sired all three young at his nesting territory (with two females) and two of three nestlings at a territory 1.9 km away.

^c^ One nest in North Dakota in 2013 and one nest in Wisconsin 2012 had both an EPP and CBP occurrence.

^d^ There were no significant inter‐year differences in proportions of: EPP young/nest within North Dakota or Wisconsin (*X* ^2^_3_ = 1.84, *p* = .61; *X* ^2^_3_ = 1.23, *p* = .75, respectively), CBP young/nest (*X* ^2^_3_ = 3.51, *p* = .32; *X* ^2^_3_ = 6.89, *p* = .08), EPP nests (*X* ^2^_3_ = 0.81, *p* = .96; *X* ^2^_3_ = 0.61, *p* = .89), CBP nests (Fisher statistic = 2.19, *df* = 3, *p* = .53; Fisher statistic = 1.94, *df* = 3, *p* = .58), and EP nests (*X* ^2^_3_ = 0.43, *p* = .93; *X* ^2^_3_ = 1.49, *p* = .68).

### 2.3 Laboratory techniques

We assayed the same six microsatellite loci (AgCA222, Takaki et al., 2009; Age7.1JT, Topinka & May, 2004, Sonsthagen et al., 2012; BV13 and BV20; Gautschi et al., 2000; NVH206, and NVH195‐2, Nesje & RØed, 2000) as Rosenfield et al. (2015), with the number of alleles ranging from 5 to 22 alleles/locus and observed heterozygosity ranging from 0.538 to 0.895 (see Table 2 in Rosenfield et al., 2015). No evidence of allelic dropout, null alleles, or scoring errors was detected across markers with Micro‐Checker (van Oosterhout et al. 2004, see Rosenfield et al., 2015). Overall probabilities of identities were 1.288 × 10^−8^ (i.e., 1 in 77,639,752 individuals with the same genotype) within a randomly mating population (PID) and 2.035 × 10^−3^ (i.e., 1 in 491) among siblings (PIDsib); probabilities of non‐exclusion (PIDnex) were 1.413 × 10^−3^ (1 in 708) for the first parent and 1.248 × 10^−3^ (1 in 801) for the second parent (see Rosenfield et al., 2015). Denominator values calculated for PID, PIDsib, and PIDnex are greater than the estimated census size of Cooper's Hawks in the United States and Canada (1,000,000; PIF, 2021) and WI (15,000 breeding adults; Bielefeldt et al., 1998). Data collection and processing followed Sonsthagen et al. (2004). Briefly, genomic data was extracted using the procedure described by Medrano et al. (1990) with modifications described in Sonsthagen et al. (2004). Genomic data was quantified with fluorometry, diluted to 50 ng mL^−1^ working solutions, and microsatellite genotype data collected at the six loci (listed above). Putative family groups with an extra‐pair fertilization occurrence were re‐extracted and genotyped at loci with mismatches among putative family members for 10 replicates. No inconsistencies in non‐matching genotypes were observed across multiple DNA extractions and PCR amplifications.

**TABLE 2:** Brood sizes, number of sampled nests (*N*), number of extra‐pair (EP) young, number of extra‐pair paternity (EPP) young, number of conspecific broad parasitism (CBP) young, and total number of young for each brood size for Cooper's Hawks breeding in British Columbia (2011), North Dakota (2012–2015), and Wisconsin (2012–2015).

Brood size	*N*	EP^a^	EPP^a^	CBP^a^	Number of young
British Columbia					
1	1	1 (100.0)	0 (0.0)	1 (100.0)	1
2	2	0 (0.0)	0 (0.0)	0 (0.0)	4
3	6	6 (33.3)	2 (11.1)	4 (22.2)	18
4	6	7 (29.2)	2 (8.3)	5 (20.8)	24
5	4	4 (20.0)	4 (20.0)	0 (0.0)	20
6	0	0 (0.0)	0 (0.0)	0 (0.0)	0
North Dakota					
1	3	1 (33.3)	1 (33.3)	0 (0.0)	3
2	9	2 (11.1)	1 (5.6)	1 (5.6)	18
3	10	7 (23.3)	6 (20.0)	1 (3.3)	30
4	14	14 (25.0)	10(17.9)	4 (7.1)	56
5	17	18 (21.2)	9 (10.6)	9 (10.6)	85
6	0	0 (0.0)	0 (0.0)	0 (0.0)	0
Wisconsin					
1	1	0 (0.0)	0 (0.0)	0 (0.0)	1
2	3	2 (33.3)	2 (33.3)	0 (0.0)	6
3	14	10 (23.8)	7 (16.7)	3 (7.1)	42
4	15	7 (11.7)	3 (5.0)	4 (6.7)	60
5	14	17 (24.3)	12 (17.1)	5 (7.1)	70
6	1	0 (0.0)	0 (0.0)	0 (0.0)	6

*Note*: Percent of EP, EPP, and CBP young for each brood number and by site are in parentheticals.

^a^ There were no significant differences in proportions of EP, EPP, and CBP young among brood sizes in British Columbia (*X* ^2^_4_ = 2.61, *p* = .63; Fisher Statistic = 2.05, *df* = 4, *p* = .73; Fisher Statistic = 7.08, *df* = 4, *p* = .13, respectively), North Dakota (*X* ^2^_4_ = 1.23, *p* = .87; *X* ^2^_4_ = 3.25, *p* = .52; Fisher statistic = 1.47, *df* = 4, *p* = .83), or Wisconsin (*X* ^2^_5_ = 4.49, *p* = .48; Fisher statistic = 6.91, *df* = 5, *p* = .23; Fisher statistic = 1.50, *df* = 5, *p* = .91).

### 2.4 Data analysis

The program COLONY version 2.0.6.7 (Wang, 2004) was used to assign parentage jointly to identify maternal‐offspring mismatches and paternal‐offspring mismatches. Offspring with genotype mismatches at one or more loci between the putative maternal or paternal genotype were classified as CBP or EPP, respectively. All adults irrespective of year sampled and young from the previous season(s) were included in the pool of candidate parents to aid in the detection of fertilization by non‐territorial adults. Maternal and paternal genotypes assigned to offspring in COLONY were verified five times with matching scores.

Queller and Goodnight's (1989) index of relatedness (*r*_*xy*_) was calculated to infer the familial relationship between breeding pairs and among young and adults sampled at the same nest site using Identix (Belkhir et al., 2002). Pairwise *r*_*xy*_ estimates range from −1 to 1 and were interpreted as follows: *r*_*xy*_ of ≥0.45 as first‐order relationships, 0.20–0.45 as second‐order relationships, and ≤0.2 as unrelated.

We pooled EPP and CBP data from all urban (*n* = 29) and rural (*n* = 19) nests in Wisconsin because there were no statistical differences (*p* > .05) in EPP and CBP frequencies in urban nests in Oshkosh (*n* = 4/10 EPP, *n* = 0/10 CBP) vs Stevens Point (*n* = 5/19 EPP, *n* = 4/19 CBP), or between this combined urban sample (*n* = 9/29 EPP, *n* = 4/29 CBP) and rural nests (*n* = 6/19 EPP, *n* = 3/19 CBP).

We used StatXact‐Turbo (Mehta & Patel, 1992) to calculate Chi‐square and Fisher's exact probabilities in comparing proportions. We used mixed‐effects logistic regression models to determine if brood size, age of female (i.e., 1 or ≥2 years) tending the nest, and mass of adult male and (or) female tending the nest predicted occurrence of EPP or CBP. Site and year were both included as random effects. Models were fit using the glmmTMB function from the glmmTMB package (Mollie et al., 2017) in the R environment for statistical computing (R Development Core Team, 2020). Significance was assessed at *p* ≤ .05.

## 3 RESULTS

We collected multilocus genotypes for 122 adult and 444 young Cooper's Hawks from a total of 120 nests (Table 1). All individuals in our data set (including siblings) had a unique multi‐locus genotype, indicating that our suite of microsatellite loci had sufficient resolution to identify individuals and detect deviations from a monogamous mating system. Evidence of extra‐pair young (EP) from both EPP and CBP was present in all three study sites and across all years (Table 1). The total proportion of EPP young (11.9%–14.1%), CBP young (6.5%–14.9%), and nests with EP young (42.1%–45.8%) were comparatively similar and statistically non‐significant across study sites (Table 1). WI had a greater inter‐annual variation in the proportion of young resulting from EPCs (EPP 10.6%–19.4%; CBP 1.7–14.9%) compared to ND. EPP and CBP strategies combined exhibited similar and non‐significant proportions of 27%, 22%, and 19% of a total of 444 nestlings being EP young among our BC, ND, and WI study sites, respectively (*X* ^2^_2_ = 1.01, *p* = .60). Within both ND and WI, we found no significant inter‐year variation in proportions of: EPP young/nest, CBP young/nest, EPP nests, CBP nests, or EP nests (Table 1).

The proportion of EP young (0.0%–100.0%), EPP young (0.0%–33.3%), and CBP young (0.0%–100.0%) varied among brood sizes but were not significant at any of the three sites (Table 2). Interestingly, most of the extra‐pair young at BC (95.8%), ND (73.5%), and WI (68.8%) had *rxy* estimates consistent with a second‐order familial relationships with the attending adult that is not the sire or dam (Table 3).

**TABLE 3:** Familial relationships inferred by Queller and Goodnight *rxy* values among extra‐pair young and attending adult that is not the sire or dam for Cooper's Hawks nesting in British Columbia (2011), North Dakota (2012–2015), and Wisconsin (2012–2015).

	Unrelated (<0.2)	Second‐order (0.20–0.45)	First‐order (>0.45)
British Columbia			
2011	1	23	2
North Dakota			
2012	3	7	0
2013	3	12	0
2014	1	7	0
2015	3	2	0
All years	10	28	0
Wisconsin			
2012	3	5	0
2013	5	4	1
2014	2	4	1
2015	0	9	0
All years	10	22	2

*Note*: Pairwise *rxy* values assigned to the three relationships are in parentheticals.

Among all study sites, only one putative, extra‐pair sire was identified among 38 extra‐pair males: a territorial male sired all three young at his BC nest (with two females) and two of three nestlings at a territory 1.9 km away. All remaining 37 extra‐pair sires and all 33 females involved in CBP occurrences on our study sites were unsampled, apparently unpaired, non‐territorial floaters (Table 1). At nests for which we have multi‐year data (*n* = 21), no EP young were detected at 28.6% (*n* = 6) of the nests across all years with EPP and CBP detected across all years at one nest each (4.8%). Most nests (61.9%; *n* = 13/21) switched among EPP, CBP, and no EP young across years. EPP was detected at 57.1% (*n* = 12) of the nests in at least 1 year, CBP was detected at 23.8% (*n* = 5) of the nests in at least 1 year, and two nests (9.5%), one each in ND and WI, had both EPP and CBP detected across years (Table 1).

None of the possible explanatory variables included in the logistic regression models (i.e., male mass, female mass, brood size, and female age) significantly predicted the presence of EPP or CBP in nests (Table 4). The lowest probability value (0.08) occurred for male mass in both EPP and CBP nests.

**TABLE 4:** Results of mixed‐effects logistic regression models testing for relationships between reproductive indices and the occurrence of extra‐pair (EP) young due to extra‐pair paternity (EPP) and conspecific brood parasitism (CBP) in nests of Cooper's Hawks in British Columbia (BC, 2011), North Dakota (ND, 2012–2015), and Wisconsin (WI, 2012–2015) using site and year as random effects.

EPP					CBP				
	Estimate	Std err	*z* value	*p*		Estimate	Std err	*z* value	*p*
Intercept	−8.58	6.11	−1.4	.16	Intercept	5.49	5.1	1.08	.28
Male Mass^a^	0.03	0.02	1.73	.08	Male Mass^a^	−0.03	0.02	−1.73	.08
Female Mass^b^	−0.01	0.01	−0.81	.42	Female Mass^b^	0.005	0.88	0.53	.6
Brood Size	−0.03	0.26	−0.13	.9	Brood Size	−0.02	0.31	−0.05	.96
Female Age^c^	0.64	0.92	0.7	.49	Female Age^c^	−0.58	0.84	−0.69	.49

*Note*: None of the covariates of male mass, female mass, brood size, or female age (one or ≥2 years of age) were significantly related to the occurrence of EP young in nests with EPP or CBP.

^a^ Mean male mass (*n*) at EPP nests, CBP nests, and nests with no EP young was 312 g (14), 292 g (6), and 292 g (27) for BC; 321 g (8), 304 g (3), and 316 g (19) for ND; and 331 g (2), 324 g (5), and 333 g (10) for Wisconsin, respectively.

^b^ Mean female mass (*n*) at EPP nests, CBP nests, and nests with no EP young was 500 g (2), 496 g (4), and 528 g (10) for BC; 554 g (n), 557 g (6), and 552 g (21) for ND; and 586 g (9), 597 g (7), and 582 g (25) for WI, respectively.

^c^ One year old females occurred in: 2 of 3 (67%) EPP nests, 3 of 5 (60%) CBP nests, and 4 of 11 (36%) nests with no EPY; and 1 of 16 (6%) EPP nests, 2 of 8 (25%) CBP nests, and 6 of 30 (20%) nests with no EPY; and 3 of 15 (20%) EPP nests, 1 of 7 (14%) CBP nests, and 4 of 27 (15%) nests with no EPY in BC, ND, and WI, respectively. One nest each in ND and WI with both EPP and CBP was used twice in tallies of nests regarding female age.

## 4 DISCUSSION

We unexpectedly discovered unique, combined high rates of alternative breeding strategies in three widely spaced populations of a solitary, socially monogamous nesting raptor. We also discovered CBP in all study years and in all three populations, a strategy previously unknown to occur in solitary breeding raptors. We note that our results indicate a specific form of CBP, quasi‐parasitism (Magonyi et al., 2021), as all EP young due to CBP did not involve DNA from extra‐pair males. EPP and CBP occurred in 16%–31% and 15–26% of total nests, respectively (Table 1). Despite the high co‐occurrence of extra‐pair young, EPP and CBP rarely appeared in the same brood, as both strategies were found in only 4% of the total 54 nests with EP young. EP young from EPP and CBP in aggregate occurred in nearly half (42%–46%) of all nests among our three populations and thus combined (45%) these strategies appear nearly equivalent to the proportion of genetically monogamous Cooper's Hawk nests (Table 1). These combined metrics exceed the earlier finding of EP young exclusively from EPP in 34% of 44 Cooper's Hawk nests in Milwaukee, WI, and are ~2–4x higher than found in any other raptor, including the colonial nesting Lesser Kestrel and Red‐footed Falcon, the only other raptors reported to exhibit CBP (Costanzo et al., 2020; Magonyi et al., 2021).

We found that 12%–14% and 7%–15% of all nestling Cooper's Hawks in each of our three study populations were EP young from EPP and CBP, respectively (Table 1). These strategies combined resulted in non‐significant proportions of 27%, 22%, and 19% of a total of 444 nestlings being EP young among our BC, ND, and WI study sites, respectively. Excluding an earlier report of 19% EP young exclusively from EPP in the Milwaukee site (Rosenfield et al., 2015), the percentage of EP young from combined alternative reproductive strategies in BC, ND, and WI (22%) resulted in higher frequencies of EPY than reported in other studies of raptors, including the two colonial nesting falcons (0.0%–11.2%; Griffith et al., 2002, Costanzo et al., 2020, Magonyi et al., 2021).

In our study, demographic and life history drivers appeared prescient (sensu Brouwer & Griffith, 2019, Lyon & Eadie, 2008) based on the hypothesis that the high rate (34%) of EPP per nest in Milwaukee results from a synergy of the strong association (not found in other raptors) between courtship feeding and copulations (wherein coitus virtually always “immediately” follows prey deliveries by males and while the female is feeding), and a high nesting density in a food‐rich setting (Rosenfield et al., 2015). High nesting densities should increase encounter rates involving extra‐pair birds (Mayer & Pasinelli, 2013). Indeed, we predicted and found relatively similar, high, and non‐significant differences in EPP frequencies per nest of 16% (BC), 30% (ND), and 31% (WI) among populations of Cooper's Hawks where high breeding densities, both urban and rural, were similar to the Milwaukee population. Notably, BC, ND, and WI populations (including Milwaukee) exhibit the highest reproductive indices (i.e., ~3.6 young/nest) for the species, therefore suggesting ample prey populations (Rosenfield et al., 2020).

Interestingly, while EPP rates per nest were higher than CBP frequencies in both ND (30% EPP vs. 15% CBP) and WI (31% EPP vs. 15% CBP), CBP frequencies, although non‐significant among sites, were higher than EPP rates in BC (EPP 16% vs CBP 26%; Table 1). What drives the comparative higher CBP rate in BC is unknown, but we speculate that ocean water surrounding the BC study site acts as a barrier limiting off‐city dispersal of individuals, especially females, who typically disperse farther than males from natal sites (Rosenfield et al., 2020). Notably, this urban site exhibits the highest documented proportion of breeding yearling female Cooper's Hawks in North America; indeed, in some years almost 50% of nesting females are yearlings (Rosenfield, 2018). Earlier documented was this insular site's higher potential for philopatry and associated records of close inbreeding among putative sibling Cooper's Hawks (Stewart et al., 2007). Similarly, we note that we had only a single detection of an “unrelated” (*rxy* ≤0.2) familial relationship for BC, which is markedly lower than observations at ND and WI (see Table 3). It is therefore conceivable that limited dispersal off‐island increases the number of females locally, and hence drives a higher proportion of non‐dispersed birds into brood parasitism relative to WI and ND.

Although body mass and age are correlated with various aspects of breeding in Cooper's Hawks (Rosenfield et al., 2015), we, as predicted, found no significant relationship between these traits and rates of EPP and CBP per nest. But contrary to our prediction, there was no relationship between EPP and brood size among BC, ND, and WI populations (Tables 2 and 4), and thus no discernible support for the hypothesis (Rosenfield et al., 2015) that territorial females traded copulations for food to maximize energy intake for increased production. However, it is conceivable that courtship feeding by extra‐pair males may contribute food requisite for reproductive success and (or) sustenance during the 30 days pre‐incubation period (Rosenfield, 2018). Indeed, we speculate that territorial females might accept or even solicit (via vocalizations) EPCs for prey (Rosenfield, 2018).

Regardless of the relevant biological correlates regarding the presence and frequency of EPP and CBP in nests, and despite the risk or putative fitness costs of raising unrelated young (Yom‐Tov, 1980), territorial adult Cooper's Hawks in our study populations did not, as in Milwaukee, appear to reduce their parental investment because our productivity indices are among the highest for the species and thus extra‐pair adults attain fitness via alternative reproductive strategies (Rosenfield et al., 2015, 2020).

As per our prediction, extra‐pair males were mostly unsampled, unobserved, and likely unpaired birds. Indeed, we identified only one captured extra‐pair sire among 38 extra‐pair males, and none of the 33 parasitic females was captured. Similarly, extra‐pair sires were caught at only 2 (13%) of 15 Cooper's Hawk nests in the Milwaukee study (Rosenfield et al., 2015). Except for a few instances in WI (see below) and ND regarding observed EPCs during the pre‐incubation period among territorial breeders and occasional instances of yearling, apparently floater males near nests with young in Wisconsin, observations of extra‐pair interactions involving Cooper's Hawks during the pre‐nestling stages are rare on these study sites (Rosenfield, 2018; Rosenfield et al., 2016). Given the high proportion of territories sampled, and that we capture or identify via color bands ≥90% of territorial males and females each year in all three study sites, we contend, as with the Milwaukee study, that most extra‐pair adults were floaters.

The absence of CBP in Milwaukee relative to its presence in all study years and populations in this study suggests that this strategy is conditional. Territories were available to prospecting females in the colonizing Milwaukee breeding population because it was growing during that study (Stout & Rosenfield, 2010). Conversely, BC, ND, and WI had stable nesting populations (Rosenfield, 2018) with few available breeding sites for prospecting females. Notably, floater populations are larger in stable breeding populations of birds (Hunt, 1998; Moreno, 2016). Thus, conceivably many females in our three study sites were relegated to being floaters and some became parasites. Alternative explanations, such as parasitizing after nest failures or being a lifelong specialist parasite (Lyon & Eadie, 2008) seem less applicable to the absence of CBP in Milwaukee because ~10%–20% of our nests in all study sites fail each year (Rosenfield et al., 2020). Yet no CBP was found in Milwaukee in three study years, and all parasitic females in this study were different individuals across years in ND and WI, as well as in BC in 1 year.

Notably and post design to this study, there has been a recent increase in records of extra‐territorial visits involving prospecting females, and courting involving floaters and paired birds of both sexes with territorial residents during the pre‐incubation and incubation periods across the breeding range of Cooper's Hawks (e.g., Deal et al., 2017; Maione, 2024). Similarly, we documented behavioral interactions of at least three different females at one WI territory across three weeks prior to incubation (Rosenfield et al., 2016). And in another report, and despite aggressive posturing by an older, incubating female, an intruding, apparent floater yearling female usurped this older female's nest in Ontario, Canada (Maione, 2024). Ellis and Depner (1979) speculated that more than one female may have laid eggs in an over‐sized clutch of seven eggs at another Ontario nest. These reports and findings in this study augment the suggestion by Rosenfield et al. (2016) that some of the social dynamics of nesting Cooper's Hawks are complex across their breeding range.

Our novel discovery of co‐occurring, relatively high rates of EPP and CBP among populations indicates that alternative reproductive strategies are common and ergo an important and possibly evolutive aspect of Cooper's Hawk's reproduction. However, we could not link select co‐variates to alternative strategies. Moreover, our surprising detection of CBP in a solitary nesting raptor and the virtual absence of data on the traits of extra‐pair adults, neither captured nor observed, hamper our understanding of selective forces influencing the inter‐population presence of these reproductive strategies—strategies that deviate markedly from most other raptors. Regardless, raptors are poorly represented in taxa‐wide reviews of alternative reproductive strategies in birds and other animals, and thus, our surprising discoveries importantly enhance our knowledge of the extent and diversity of these strategies among groups of animal species.

## AUTHOR CONTRIBUTIONS

**Robert N. Rosenfield:** Conceptualization (lead); data curation (equal); formal analysis (equal); funding acquisition (equal); investigation (equal); methodology (equal); project administration (equal); resources (equal); software (equal); supervision (equal); validation (equal); visualization (equal); writing – original draft (equal); writing – review and editing (equal). **Sarah A. Sonsthagen:** Conceptualization (lead); data curation (equal); formal analysis (equal); funding acquisition (equal); investigation (equal); methodology (equal); project administration (equal); software (equal); supervision (equal); validation (equal); visualization (equal); writing – original draft (equal); writing – review and editing (equal). **William E. Stout:** Conceptualization (supporting); data curation (supporting); formal analysis (supporting); funding acquisition (lead); investigation (lead); methodology (supporting); project administration (supporting); software (supporting); supervision (lead); validation (lead); visualization (lead); writing – original draft (lead); writing – review and editing (lead). **Timothy G. Driscoll:** Conceptualization (supporting); data curation (supporting); formal analysis (supporting); funding acquisition (lead); investigation (lead); methodology (supporting); project administration (supporting); resources (lead); software (supporting); supervision (supporting); validation (equal); visualization (supporting); writing – original draft (supporting); writing – review and editing (lead). **Andrew C. Stewart:** Conceptualization (supporting); data curation (supporting); formal analysis (supporting); funding acquisition (lead); investigation (lead); methodology (supporting); project administration (supporting); resources (lead); software (supporting); supervision (supporting); validation (lead); visualization (supporting); writing – original draft (supporting); writing – review and editing (supporting). **Paul N. Frater:** Conceptualization (supporting); data curation (lead); formal analysis (equal); funding acquisition (supporting); investigation (lead); methodology (lead); project administration (supporting); resources (equal); software (equal); supervision (supporting); validation (lead); visualization (lead); writing – original draft (lead); writing – review and editing (lead). **Sandra L. Talbot:** Conceptualization (lead); data curation (equal); formal analysis (equal); funding acquisition (equal); investigation (equal); methodology (equal); project administration (equal); resources (equal); software (equal); supervision (equal); validation (equal); visualization (equal); writing – original draft (supporting); writing – review and editing (supporting).

## CONFLICT OF INTEREST STATEMENT

The authors declare no conflict of interest that influenced the conduct or presentation of this study. Any use of trade, product, or firm names is for descriptive purposes only and does not imply endorsement by the U.S. Government.

## Data Availability

Genotype information collected with funds provided by the U.S. Geological Survey are available from U.S. Geological Survey, Alaska Science Center Data Repository https://doi.org/10.5066/P914SGB1 (Sonsthagen & Talbot, 2020). Data on nests, nestlings, and nesting adults, and R scripts are available at Harvard DataVerse: https://dataverse.harvard.edu/privateurl.xhtml?token=65d40659‐e59b‐4a0c‐887f‐7a22eab7ada1
